# Plant Reproductive Genomics at the Plant and Animal Genome Conference

**DOI:** 10.1002/cfg.469

**Published:** 2005-04

**Authors:** Jim Leebens-Mack, Douglas E. Soltis, Pamela S. Soltis

**Affiliations:** 1 Department of Biology and Huck Institutes of the Life Sciences Pennsylvania State University University Park PA 16802 USA; 2 Department of Botany University of Florida Gainesville FL 32611 USA; 3 Florida Museum of Natural History University of Florida Gainesville FL 32611 USA


**Comparative and Functional Genomics**
*Comp Funct Genom* 2005; **6**: 159–169.
Published online in Wiley InterScience (www.interscience.wiley.com). **DOI:** 10.1002/cfg.469

**Conference Paper**

### Plant reproductive genomics at the Plant and Animal Genome Conference

Jim Leebens-Mack[1]*, Douglas E. Soltis[2] and Pamela S. Soltis[3]
[1] *Department of Biology and Huck Institutes of the Life Sciences, Pennsylvania State University, University Park, PA 16802, USA*
[2] *Department of Botany, University of Florida, Gainesville, FL 32611, USA*
[3] *Florida Museum of Natural History, University of Florida, Gainesville, FL 32611, USA*

**Correspondence to:*
*Jim Leebens-Mack, Department of Biology and Huck Institutes of the Life Sciences, Pennsylvania State University, University Park, PA 16802, USA.*
*E-mail: jleebensmack@psu.edu*

Received: 31 January 2005
Accepted: 8 February 2005

**Keywords:** **angiosperms; floral development; ABC model; comparative method**

### Introduction

Exciting advances in our understanding of plant reproductive biology are being spurred both by new research on established model systems and by the emergence of new models. Moreover, comparative analyses are placing knowledge derived from each of these systems into an evolutionary framework and thus providing insights into the processes responsible for the diversity of forms we see today. The number of species with large EST sets for floral transcriptomes is growing rapidly (Figure 1; **http://pgn.cornell.edu/**) and expression profiling of tagged genes is under way for many of these species. While there are still few tractable systems for experimental investigations of gene function, the development and refinement of virus-induced gene silencing (VIGS; reviewed in Benedito *et al.*, 2004; Lu *et al.*, 2004; Robertson, 2004) and targeting-induced local lesions genomes (TILLING; reviewed in Henikoff *et al.*, 2004; Stemple, 2004) promise to expand the application of reverse genetics to non-model species. Experimental analysis in a broader range of plant lineages (Figure 1), including the basal-most eudicot lineages (Ranunculales), non-grass monocots, magnoliids and the basal-most angiosperm lineages (Amborellaceae and Nymphaeaceae; see Angiosperm Phylogeny Group, 2003), will help us understand how gene function has evolved since the most recent common ancestor of *Arabidopsis* and grain models (rice, maize, wheat and others) and all angiosperms.

The **Plant Reproductive Genomics Workshop** at the Plant Animal Genome Conference was organized to present recent advances in our comprehension of floral development and discuss how new genomic resources for an expanding set of plant species is continuing to deepen this understanding. The main topics covered in the workshop this year were floral diversity, variations of the classical ABC model of floral development, genetic analysis of reproductive traits, the regulation of flowering time and the origin and characterization of the floral transcriptome in the most recent common ancestor of all extant angiosperms. These topics were covered by 13 speakers in this workshop and by a number of speakers in other workshops at PAG XIII. The papers presented in the Plant Reproductive Genomics Workshop will be included in a symposium volume to be published early next year in the *Advances in Botanical*

Copyright © 2005 John Wiley & Sons, Ltd.

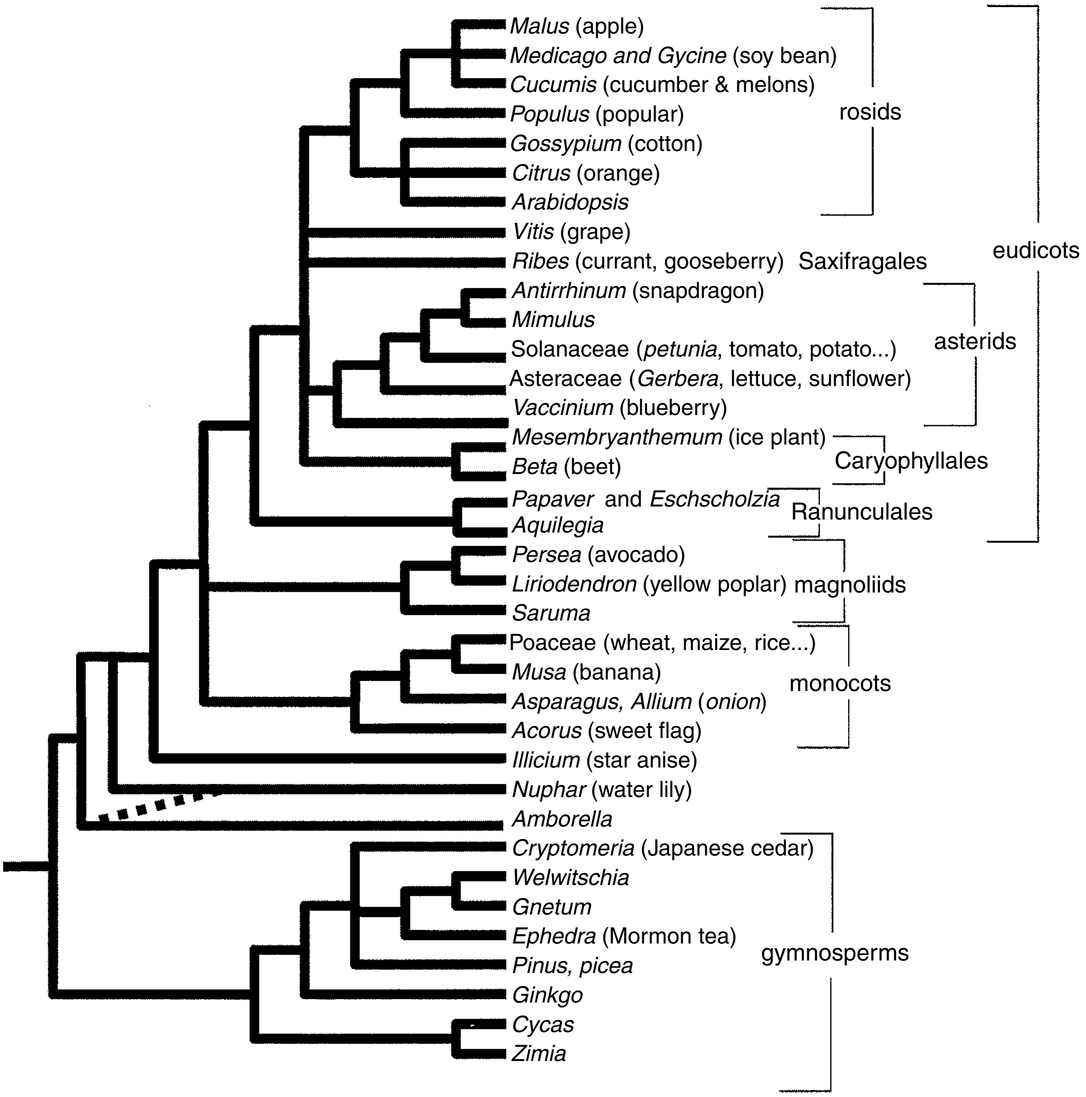

**Figure 1.** Expressed sequence tags (ESTs) are being generated for a growing number of plant species representing major lineages of angiosperms and gymnosperms. The phylogeny shows the relationships among species for which large EST sets are available now or will be available in the near future. Multigene phylogenetic analyses provide varying levels of support for *Amborella* alone as sister to all other angiosperms (as indicated by the dashed line) or *Amborella* plus waterlilies, forming a clade that is sister to all other extant angiosperms (Zanis *et al.*, 2002; Stefanovic *et al.*, 2004)

*Research* series (Elsevier/Academic Press). Here we highlight some of the points presented in these talks.

### Floral diversity

The current models used for studies of floral development exhibit a small fraction of the diversity of floral forms observed across the angiosperms. **Peter Endress** (University of Zurich) characterized the diversity of ontogenetic programs observed in floral development. Both *Arabidopsis* and grass flowers have their organs developing in well-defined whorls, whereas many species in the basal-most angiosperm lineages exhibit a spiral organization of floral organs with sequential, spiral development of perianth parts, then male organs, and finally female organs (Endress, 2001). This basic difference in phyllotaxy has implications for other aspects of floral development, e.g. the

Copyright © 2005 John Wiley & Sons, Ltd. 

number of organs in a whorl may be limited by crowding. Synorganization of parts, as observed for eudicot species with fused corollas or syncarpous ovaries, may be achieved by congenital fusion in species with whorled floral organization, but this is very rare in species with spirally arranged floral organs. Postgenital coherence was important for the origin of angiospermy, as the carpel was probably sealed by secretion in the earliest angiosperms. Postgenital coherence through cuticular interdigitation, cellular interdigitation and postgenital fusion has also been important in the evolution of synorganization throughout angiosperm history, and these processes deserve more intensive investigation in diverse species with contrasting floral forms.

A striking variation on the development of flowers with whorled phyllotaxy is the existence of a ring meristem in some non-model lineages. The ring meristem is associated with a developmental decoupling of whorls with male and female organs. This decoupling has in turn extended the time of stamen initiation and allowed for the development of different types of stamens in some lineages. In the case of taxa such as *Dillenia* (Dilleniaceae), stamens are initiated centrifugally even after the gynoecium is completely closed, and sterile stamens formed later in development may act to increase pollinator activity (Endress, 1997). Again, this mode of floral development is not observed in current model systems and investigations of the changes in the molecular regulation of floral development in these systems would provide insights into the evolution of novel pollination systems.

### Variations on the ABC(DE) model of floral organ initiation

Over the last 15 years, forward and reverse genetic investigations focused largely on the core eudicots *Antirrhinum majus, Arabidopsis thaliana* and *Petunia hybrida* have formed a firm foundation for our understanding of the molecular mechanisms regulating flower development (reviewed in Irish, 2003; Jack, 2004; Kramer and Hall, 2005). In the early 1990s ground-breaking synthetic analyses of work on floral homeotic mutants in *Antirrhinum* and *Arabidopsis* culminated in the ABC model of floral development (Schwartz-Sommer *et al.*, 1990; Coen and Meyerowitz, 1991). In this model (Figure 2), A-class genes specify development of sepals (first whorl) and petals (second whorl); B-class genes specify development of petals and stamens (third whorl); and C-class genes specify development of stamens and carpels (fourth whorl). The model also stipulates that A-class and C-class genes negatively regulate each other. Later experiments showed that a fourth class of genes is necessary for ovule development (Columbo *et al.*, 1995), and a fifth class (E-class) works with the ABC genes to regulate floral organ initiation (Pelaz *et al.*, 2000). All but one (*AP2*) of the genes implicated in the ABC(DE) model of floral development are transcription factors in the MIKC-type MADS box gene family (Figure 2). According to the quartet model (Theissen and Saedler, 2001), specific combinations of A-, B-, C- and E-class MADS proteins are hypothesized to form heterodimers or higher-order complexes that bind to CArG-box DNA motifs and regulate the development of specific floral organs (Theissen and Saedler, 2001).

Soon after the ABC model was proposed, evolutionary biologists started to reconstruct the relationships among MADS box genes (Doyle, 1994; Purugganan *et al.*, 1995). This work has continued as more MADS genes have been identified (reviewed in Becker and Theissen, 2003) and comparative analyses are providing a deep understanding of how gene expression patterns and gene function have evolved through angiosperm history. A number of the speakers presented their latest

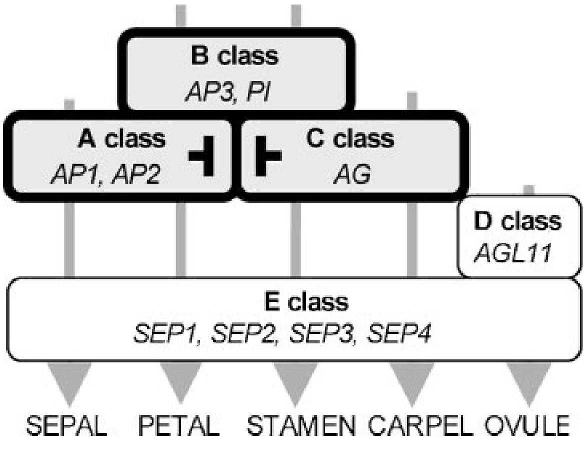

**Figure 2.** In the ABC(DE) model of genetic control over floral development, distinct classes of genes specify organs in whorls 1, 2, 3 and 4 of the flower and the ovules. In addition, class A and class C genes regulate each other antagonistically. The identities of these genes in *Arabidopsis* are shown in for each functional class

Copyright © 2005 John Wiley & Sons, Ltd.

research on MADS box gene expression and function. Gene duplication and shifting function were common themes in these talks. Aspects of the ABC(DE) model developed for the core eudicots seem to hold across all of the angiosperms, but the evolution of gene expression, and presumably function, is quite dynamic. Moreover, in many basal lineages of flowering plants, as well as basal eudicots, the ABC model has to be revised to accommodate flowers with spiral phyllotaxy or an undifferentiated perianth, rather than distinct sepals and petals.

**Kerstin Kaufmann**, a student in Guenter Theissen's lab (Friedrich Schiller University, Jena), presented their work on the physiochemical basis of the regulatory network of floral development hypothesized in the quartet model. The interaction of B-class genes regulating development of the petals and stamens has been studied most intensively in yeast two-hybrid experiments. In angiosperms, the B-class genes fall into two well-supported clades, designated *DEF* and *GLO* after the *Antirrhinum* gene placed in each clade with the *Arabidopsis* genes *AP3* and *PI*, respectively. The *DEF* and *GLO* clades arose from a common ancestor following a gene duplication that occurred some time after the divergence of the lineages leading to extant angiosperms and gymnosperms (Winter *et al.*, 2002; Stellari *et al.*, 2004; Kim *et al.*, 2004). Protein interaction experiments and functional studies have shown that B-class function in most angiosperms studied to date requires both *DEF* and *GLO* orthologueues, which form a heterodimer in yeast two-hybrid experiments. The finding that the most closely related B-type gene in *Gnetum* (*GGM2*) is able to function as a homodimer led to the conclusion that obligate heterodimerization of B-type genes arose after the *DEF/GLO* duplication (Winter *et al.*, 2002). Recent yeast two-hybrid experiments, however, have shown that *GGM2* is able to form heterodimers with several other MIKC-type MADS gene products identified in *Gnetum*. This raises the question of whether the ability of ancestral B-class MADS proteins to form heterodimers was a precursor to obligate heterodimerization of MADS proteins for B-function in flowering plants.

**Brendan Davies** (University of Leeds) contrasted the function of C-class genes in *Antirrhinum* and *Arabidopsis* and touched on the utility of new genomic resources for studies of gene function in *Antirrhinum*. A gene duplication early in the history of core eudicots, before the divergence of rosids (including *Arabidopsis*) and asterids (including *Antirrhinum*), gave rise to two clades with genes that have been retained in both lineages. Davies and collaborators are comparing BAC clones containing the two *Antirrhinum* genes PLENA (PLE) and FARINELLI (FAR) with homologous regions in the *Arabidopsis* genome anchored by SHATTERPROOF1 (SHP1) and SHP2 and the C-class gene AGAMOUS (AG). Syntenic patterns are predicted based on recent phylogenetic analyses identifying AG and FAR as orthologues; and *SHP1* and *SHP2* are recent duplicates that are orthologues of *PLE* (Kramer *et al.*, 2004).

The relationships among AG homologues in *Arabidopsis* and *Antirrhinum* somewhat surprising because the phenotypes of *ag* and *ple* mutants are more similar to each other than they are to *far* and *shp1 shp2* mutants, respectively. The *ag* and *ple* mutants show homeotic conversion of reproductive tissues to perianth-like tissues, whereas *far* mutants form anthers with sterile pollen and intact carpels. The most apparent phenotype of *shp1 shp2* double mutants is indehiscent fruits, although the *SHP* genes are also expressed in ovules. Analyses of *far* and *ple* single and double mutants suggest that *PLE* is an upstream regulator of *FAR* and *FAR* is a repressor of *PLE* (Davies *et al.*, 1999). As might be predicted, based on the phenotypes of *far* and *ple*, transformation experiments showed that *FAR* expression has a greater influence on male function, while *PLE* has a stronger influence on female function. Taken together, the data on C-type gene expression and function demonstrate different patterns of subfunctionalization and perhaps neofunctionalization (*sensu* Force *et al.*, 1999) in *Arabidopsis* and *Antirrhinum* following a gene duplication event in a common ancestor.

**Toby Kellogg** (University of Missouri, St. Louis, MO) showed data on independent MADS gene duplication events in the grasses (Poaceae). Much of the work Kellogg and collaborators are doing on MADS gene evolution in the grasses is aimed at elucidating how gene duplications lead to functional and ultimately morphological diversification. Duplications of A-, C- and E-class genes just prior to diversification of the grass family have been followed by divergence in expression patterns. In all of these cases, however, duplicate genes have evolved under similar levels of constraint. There

Copyright © 2005 John Wiley & Sons, Ltd. 

has been no detectable change in the ratio of non-synonymous to synonymous nucleotide substitution rates following gene duplication.

Phylogenetic analysis of *SEPALLATA*-like (*SEP*) MADS genes shows independent duplication events in the monocots and eudicots. Whereas the E-function of *SEP1, SEP2* and *SEP3* seems largely redundant in *Arabidopsis*, expression patterns vary among many duplicates in the grass family. Investigations of one of these duplicates show that differences in the expression patterns observed among distinct grass lineages are associated with differences in spikelet development. *LEAFY HULL STERILE1* (*LHS1*) orthologues are expressed in the reproductive meristem of all grasses. However, as development proceeds in panicoid species (e.g. maize, sorghum, pearl millet) with two flowers per spikelet and top-down maturation, *LHS1*-like gene expression is limited to the upper floret. While the same pattern of expression is observed in development of the three-flowered rice spikelets, lineages with many flowers per spikelet and bottom-up maturation show gradual reduction in *LHS1*-like gene expression through development of all florets (Malcomber and Kellogg, 2004). Adding to this diversity, organ-specific expression patterns within flowers vary among species with both top-down and bottom-up spikelet development. The independent diversification of multiple MADS gene lineages early in the history of the Poaceae raises the possibility of manifold diversification in gene interaction partnerships and function. Perhaps this diversity at the molecular level has played a role in generating morphological variation and the impressive species diversity observed in the grass family.

The number of grass species is topped only by the species richness observed within the Orchidaceae and Asteraceae. **Teemu Teeri** (University of Helsinki) presented the work he and his collaborators have done on MADS gene function in *Gerbera hybrida* (Asteraceae). Like grasses, members of the sunflower family have flowers that are quite distinct from those of *Arabidopsis, Antirrhinum* or *Petunia*. The flower head (inflorescence) of *Gerbera* includes ray flowers with aborted stamens and disk flowers with reduced petals, and all flowers have specialized pappus bristles derived from sepals, fused petals and anthers, and inferior ovaries.

The B- and C-function homologues in *Gerbera* are placed with their *Antirrhinum* and *Arabidopsis* counterparts in MADS gene phylogenies. The *Gerbera SEP*-like genes, however, have very diverse functions (Uimari *et al.*, 2004). Unlike the redundant expression patterns of *SEP1, SEP2* and *SEP3* in whorls 2, 3 and 4 of *Arabidopsis* flowers, distinct *Gerbera SEP* homologues show evidence of subfunctionalization. Expression of *GRCD1* is required for stamen development, while *GRCD2* is needed for proper initiation of carpels. Downregulation of *GRCD2* also results in floral reversion, producing flowers within the carpels! In addition, reduced *GRCD2* expression leads to larger inflorescences (capitula). These observations indicate that *GRCD2*, like *SEP4* in *Arabidopsis* (Ditta *et al.*, 2004), is involved in the regulation of reproductive meristem determinacy.

**Vivian Irish** (Yale University) and co-workers have initiated analyses to examine the extent to which gene duplication, regulatory diversification and differences in protein interactions have been important in modifying the roles of MADS box genes during the evolution of flowering plants. It is noteworthy that the occurrence of several key duplications of floral organ identity genes seems to correspond with the origin of core eudicots (e.g. Irish, 2003; Litt and Irish, 2003; Kramer *et al.*, 2004; Stellari *et al.*, 2004; Kim *et al.*, 2004; Zahn *et al.*, 2005).

In efforts to attain a more complete understanding of the role of duplicated MADS genes, the Irish lab has developed a protocol for rapidly identifying all MADS box genes from a given species. Reconstruction of phylogenetic relationships among MADS genes is providing a historical context for the results of functional analyses. For example, tomato MADS box genes and their relationship to well characterized MADS genes from model species have been identified and current studies are elucidating the function of these genes in tomato flower and fruit development (e.g. DiMartino and Irish, in preparation).

Molecular genetic investigations have provided insights into the molecular evolution and function of various MADS duplicates. For example, considering the duplication of the B-class gene *AP3*, a divergent C-terminus motif in the euAP3 lineage appears to have arisen via a frameshift mutation (Vandenbussche *et al.*, 2003). The paleo-AP3 gene may have only specified stamen identity and not petal identity, as euAP3 does in core

Copyright © 2005 John Wiley & Sons, Ltd.

eudicots (Lamb and Irish, 2003; but see Whipple *et al*., 2004).

Irish stressed the importance of obtaining more MADS data, including functional analyses, for non-model angiosperms (similar statements were echoed later by Kramer and Soltis). Poppy is proposed as a potential new model, representing the phyogenetically pivotal basal eudicots. However, Irish *et al*. have encountered problems with transformation in poppy. As result, they have been developing alternative methods to carry out functional analyses in this and other non-model basal eudicots. One method that shows great promise in poppy is the use of VIGS using tobacco rattle virus (Liu *et al*., 2004).

**Elena Kramer** (Harvard University) continued the theme of modified ABC(DE) models to explain variation in floral form. Her presentation focused on recent work on duplicated B-function homologues in Ranunculaceae, a clade of basal eudicots that exhibits a great deal of variation in floral organization, with the intent of relating differential expression of paralogues to organ differentiation within a flower and to differences in floral structure among species. She began her presentation by asserting that, given the range of floral morphologies across the angiosperms and our currently rather limited understanding of the genes controlling perianth identity, there are multiple petal identity programs at work in different groups of angiosperms. Furthermore, some groups, notably Ranunculaceae, have an abundance of B-function homologues that are not due to increases in ploidy and that may play roles in floral differentiation in the family (e.g. Kramer *et al*., 2003). Based on her lab's work on *Aquilegia* (columbine), it appears that multiple *AP3* paralogues may each have distinct functions in perianth differentiation. For example, the *PI* homologue and one of the *AP3* homologues, *AP3-1*, have expression patterns consistent with *PI* and *AP3* in *Arabidopsis*. *AP3-2* is expressed later in the petals and for longer duration in the stamens, whereas *AP3-3* expression is restricted to petals and may play a role in the development of the spurred petals and nectaries typical of *Aquilegia*. Later expression of *PI* in the outer perianth may confer petaloid characteristics to these organs, rather than organ identity *per se*. A combination of 'sliding boundaries' (Kramer *et al*., 2003) of duplicate genes and temporal variation in expression may be responsible for perianth differentiation in *Aquilegia*. Using yeast-2-hybrid studies, Kramer found some interactions between *AP3-2* and *AP3-3*, but most heterodimers are between *PI* and *AP3* subunits. Future analysis of numerous homeotic horticultural mutants, 35 000 ESTs and microarrays should help to refine the petal identity program(s) at work in *Aquilegia* and address the possible roles of sub- and neofunctionalization of duplicate genes in shaping floral morphology.

**Pamela Soltis** (University of Florida) presented a modified ABC(DE) model of floral organ identity for basal angiosperms characterized by an undifferentiated perianth and gradual intergradation of floral organs. The model is based on studies of expression of homologues of the ABCE-function genes of *Arabidopsis* in a set of basal angiosperms: *Amborella trichopoda, Nuphar advena* and *Illicium floridanum*, representing the three basal-most lineages of extant angiosperms (Figure 1), and three representatives of the magnoliid clade, *Magnolia grandiflora, Eupomatia bennettii* (and *E. laurina*) and *Asimina longifolia*. Of these species, only *A. longifolia* has a perianth that is differentiated into distinct sepals and petals, and *Eupomatia* lacks a perianth entirely. *Antirrhinum majus* was used as a point of reference. Homologues of all classes of ABC(DE) genes have been reported from basal angiosperms (e.g. Kim *et al*., 2004, 2005; Kramer *et al*., 1998, 2003; Litt and Irish, 2003; Stellari *et al*., 2004), and Soltis reported that most classes of these genes have been found in the species chosen for study. Using a combination of relative quantitative RT-PCR, real-time PCR and *in situ* hybridization studies, she reported consistent patterns of gene expression for floral MADS box homologues of *AP1, AP3, PI, AG* and *SEP*, despite differences in sensitivities of the techniques and in stages of floral development assayed. In general, floral MADS box genes are expressed in organs predicted by the ABC(DE) model of *Arabidopsis* and *Antirrhinum*, but expression, particularly of B-function homologues, is broader than predicted. The sole exception to this pattern is *A. longifolia*, with its sepaloid outer perianth whorl and two inner whorls of petaloid perianth parts, in which the *PI* homologue is restricted to petals and stamens and the *AP3* homologue is expressed at only low levels in the sepals. This pattern of expression is very similar to that of core eudicots characterized by a differentiated, whorled perianth and suggests

Copyright © 2005 John Wiley & Sons, Ltd. 

that the same mechanism that evolved in eudicots appears to have arisen in this magnoliid.

Based on these results, Soltis and collaborators proposed that the ancestral angiosperms most likely exhibited broad patterns of MADS box gene expression across the floral meristem. Restriction of gene expression to more localized areas of the meristem may have led to the origin of whorled flowers with distinct sepals and petals — those flowers typical of most eudicots. Furthermore, broad — and intergrading — expression of key floral regulators corresponds to patterns of morphological intergradation of adjacent floral organs in *Amborella* (e.g. Buzgo *et al.*, 2005) and likely other species. The 'fading borders' model, in which expression of each regulator is weaker at the outer and inner edges of its zone of activity, may explain the gradual transitions of floral organs — from bracts to sepaloid perianth to petaloid perianth to stamens, staminodes and perhaps carpels — often observed in basal angiosperms.

### Quantitative genetics and floral form

**John Willis** (Duke University) reported on work he and colleagues have been doing on the *Mimulus guttatus* complex (Phrymaceae, formerly Scrophulariaceae). Adding to the quantitative genetic analyses of differences in floral traits and mating systems and the nature of the species boundary between the outcrossing *M. guttatus* and the selfing species, *M. nasutus*, large sets of BAC end sequences and ESTs are being used to increase the density of the genetic map for the *M. glutattus* complex. With its small genome size (~425 Mb/1C), growing set of genomic resources, rapid regeneration time (2 months) and morphological and ecological diversity, *Mimulus* is a strong candidate for whole genome sequencing in the near future. Genetic mapping work has already identified 24 QTLs for reproductive traits in *Mimulus* (Fishman *et al.*, 2002) and led to the localization of a region responsible for true meiotic drive in hybrid crosses (Fishman and Willis, 2004). The QTL analysis shows that many genes of small effect can influence floral form and outcrossing rate. Therefore, reverse genetics involving a few candidate genes will not provide a complete picture of the genes influencing variation in flower shape and size. Quantitative analysis of variation in floral colour patterning among *Mimulus* species is under way. All of this work is providing insights into the molecular basis of reproductive diversity within and among species.

In the **International Grass Genome Initiative Workshop**, Kellogg presented the complementary QTL and candidate gene analyses of inflorescence branching in foxtail and green millet. This work, led by **Andrew Doust** (Doust *et al.*, 2004, 2005), revealed co-localization of candidate genes with QTL influencing inflorescence branch number and density, spikelet number and bristle number. For example, a marker for the *LEAFY* orthologue mapped in close proximity to a QTL for primary branch number and density. No genomic resources other than the genetic map were available for the two millet species compared in this study, but Doust, Kellogg and collaborators were able to leverage the sequence data available for related model grain species and uncover important aspects of the genetic basis of differences in the inflorescence architectures of two millet species.

### Regulation of flowering time

**Michael Purugganan** (North Carolina State University) discussed how microevolutionary analyses of population variation in *Arabidopsis thaliana* are elucidating how natural selection across a latitudinal gradient is driving the evolution of the flowering time regulatory network. Purugganan and colleagues are in the process of testing for clinal variation in all genes known to be in the flowering time regulatory network. To date, analyses of *FLOWERING LOCUS C* (*FLC*) and active forms of the upstream regulator *FRIGIDA* (*FRI*) are exhibiting latitudinal clines in haplotype frequencies (Stinchcombe *et al.*, 2004; Caicedo *et al.*, 2004). *FLC* expression, which represses flowering, is upregulated by *FRI* and downregulated by signals from the autonomous and vernalization flowering time pathways. Significant linkage disequilibrium between *FRI* and *FLC* haplotypes, together with the results of field experiments showing significant effects on size of flowering plants for both loci and their interaction, provide strong evidence for the influence of natural selection on the epistatic interaction between *FRI* and *FLC*. This exciting research is showing how natural selection acting on the initiation of reproductive development results in intraspecific variation in the genetic interactions

Copyright © 2005 John Wiley & Sons, Ltd. 

regulating flowering time. Experimental tests of alternative hypotheses for the molecular mechanisms behind this response are under way.

New findings on the control of flowering time were also presented by **J. Chris Pires** (University of Wisconsin) in the **Brassicas Workshop** and **Masahiro Yano** (National Institute of Agrobiological Sciences, Japan) in his plenary address. Pires presented work on the segregation and expression of *FLC* genes in synthetic *Brassica* polyploids (Schranz and Osborn, 2004; Pires *et al.*, 2004). Flowering time in synthetic polyploids was strongly influenced by the source of expressed *FLC* homologues at three loci. As might be expected, based on flowering time differences between the two diploid parental species, expression of *B. oleracea* homologues was greater in late-flowering lines while *B. napus* homologues were more highly expressed in early-flowering lines. Gene expression levels were influenced by chromosomal rearrangements and epigenetic gene silencing.

*FLC* is a MADS box gene, but to date, no closely related homologues have been observed in species of Brassicaceae. The regulation of flowering time in rice differs from *Arabidopsis* and *Brassica* in this regard and many others. **Masahiro Yano** presented the large body of work from his lab on the genetics of photoperiodic control of flowering time in rice (e.g. Kojima *et al.*, 2002; Izawa *et al.*, 2003; Doi *et al.*, 2004). Comparisons of QTL maps and gene expression in nearly isogenic lines (NILs) with naturally occurring loss-of-function alleles are elucidating gene interactions with a regulatory network and the molecular basis of natural flowering time among rice lines. Phylogenies for gene families including flowering time regulators from rice and *Arabidopsis* show that gene duplications in the ancestors of both species and subsequent evolution in gene function have contributed to differences in the control of flowering time in these two species.

### Characterizing the most recent common ancestor of all living angiosperms

The evolutionary processes responsible for the origin and rapid radiation of flowering plants has long been a major question in plant evolution, famously called an 'abominable mystery' by Darwin. Throughout the 1900s, the large morphological gap between flowers and potential gymnosperm relatives and possible ancestors permitted a multitude of widely different and mutually incompatible hypotheses to be entertained. The recent consensus on relationships among basal angiosperms, and the demonstration that living gymnosperms are monophyletic (so none are particularly closely related to angiosperms), now substantially narrow the range of reasonable models of angiosperm origins. Our changing views on the origin of the flowering plants are not only influenced by prevailing views and assumptions, but a renewed appreciation of old, long-ignored developmental literature has also been important. Recent evidence from genes that control development has already led to theories that are more specific and more testable than older theories.

**Michael Frohlich** (Natural History Museum, London) discussed prospects for ongoing and future research on the origin of the flower and flowering plants. The most recent theories focus on the origin of flower bisexuality from gymnosperm ancestors that produced separate male and female structures. A recent proposal for the origin of the flower is the Mostly Male Theory (Frohlich and Parker, 2000). In brief, gymnosperms generally have two copies of the *LFY* gene, referred to as the 'needle' and 'leaf' families. The 'needle' (*NEEDLY*) paralogue ($LFY_f$) is expressed only in early-developing female reproductive structures, whereas the 'leaf' paralogue ($LFY_m$) is expressed in male reproductive structures. The *LFY* duplication in gymnosperms may have accompanied or perhaps facilitated the specialization of separate male and female reproductive structures. In the Mostly Male model, *LFY* is a trigger for the origin of the flower. Angiosperms have apparently lost the female-specifying 'needle' paralogue and retained the male-related 'leaf' paralogue. Frohlich and Parker (2000) suggested that developmental control of floral organization derives more from systems operating in the male reproductive structures of the gymnosperm ancestor of angiosperms than from the female reproductive structures; hence 'mostly male.' In the Mostly Male Theory, ovules are considered ectopic in origin on male reproductive structures. Supporting this possibility is old evidence in the literature for ectopic expression in weird places in plants, such as ovules on leaves in *Ginkgo*.

Albert *et al.* (2002) provided an alternative to the Mostly Male Theory. Their model assumes that pleiotropic interactions between $LFY_m$ and

Copyright © 2005 John Wiley & Sons, Ltd. 

*$LFY_f$* were critical for stabilizing the retention of these two genes in gymnosperms, and suggests that disruption of this delicate balance between the two *LFY* genes occurred in an ancestor of modern angiosperms. This ancestral taxon might have had unisexual flowers together on the same plant, or might have been loosely bisexual. *$LFY_f$* would then have been lost through selection for an integrated bisexual reproductive axis.

Views on morphology can also change our perspective on the origin and diversification of the flower. Frohlich provided an example based on the perianth of the water lilies *Nuphar* and *Nymphaea* (Nymphaeaceae). The distinction between sepals and petals in *Nuphar* and *Nymphaea* was traditionally based on morphology, colour differences and glands. But Frohlich showed that in water lilies, sepal vs. petal identity also depends on position, age of organ, environment (light, physical contact) and interactions among these factors; furthermore, outer organs shield inner organs and the latter become petal-like. However, aspects of 'sepalness' and 'petalness' are not fixed to organs until later in development. In fact, sepal-like vs. petal-like is a difficult distinction (the terms usually refer to whole organs) but Frohlich showed that they may refer, in basal angiosperms, to organ segments, as in *Nuphar*, where part of a single organ may look like a sepal while other parts look like a petal — this is a new twist to looking at morphology and organ identity. Distinct sepal and petal developmental programs arose early in angiosperm history but may not have been clearly anchored to individual organs in early angiosperms. This anchoring did not really arise until the evolution of eudicots — further evidence of the floral developmental flexibility of basal angiosperms.

**Claude dePamphilis** (Penn State University) and collaborators involved in the Floral Genome Project (**http://www.floralgenome.org/**) are using a combination of EST sequencing and gene expression analyses to characterize the minimal genetic toolbox for floral development that must have been present in the last common ancestor of all extant angiosperms. Two very interesting observations are coming from genomic analyses of large EST sets generated for basal angiosperms *Amborella trichopoda* and *Nuphar advena*, magnoliids *Persea americana* (avocado) and *Liriodendron tulipifera* (tulip poplar), and representatives of the basal-most monocot and eudicot lineages, *Acorus americanus* and *Eschscholzia californica*, respectively (Figure 1). Adding to a growing body of evidence that polyploidization has been common in angiosperm history, genome-wide duplication events are evident in analyses of all of the listed EST sets, with the exception of *Amborella*. At the same time, estimates of the number of genes expressed in developing flower buds do not vary appreciably across species. Despite the incomplete sampling inherent in EST sequencing projects, phylogenetic analyses of gene families, including known regulators of flowering time and floral organ identity in rice and *Arabidopsis*, showed that the last angiosperm common ancestor had a diverse set of potential regulators. The placement of basal angiosperm genes in the phylogenies with rice and *Arabidopsis* genes is helping to resolve the timing of gene duplications relative to the most recent common ancestor of monocots and eudicots.

### Comparative plant genomics

Throughout the Plant Reproductive Genomics Workshop and the entire Plant and Animal Genome Conference, many and perhaps most talks included comparative analyses that drew inferences from cross-species similarities or differences in gene content, expression or function. The increasing base of species for which these data are available is improving our ability to draw accurate conclusions about the evolutionary processes that have generated the biodiversity we observe around us. **Jim Leebens-Mack** (Pennsylvania State University) ended the Workshop with some examples of how phylogenetically-based comparative genomics provides powerful tools for learning about individual plant species or groups of related species. A comparison of *Arabidopsis, Populus, Medicago* and rice genomic sequences in regions surrounding the typically single-copy gene *LEAFY* showed that the promoter of the *Medicago* orthologue has lost a conserved regulatory motif that is conserved between *Arabidopsis* and *Populus*. Cluster analysis of the predicted rice and *Arabidopsis* proteomes reveals over 4000 single-copy *Arabidopsis* genes with no orthologues in rice (**http://www.PlantTribes.org**). **Patrick Zheng**, a postdoc in **David Openheimer's** lab (University of Florida), is analysing T-insertion mutants for

Copyright © 2005 John Wiley & Sons, Ltd. 

some of these genes and finding that reduced-function mutants have highly compromised phenotypes. Orthologues of these genes were found in some of the Floral Genome Project EST sets, including the monocots asparagus and yucca, but not in the massive EST database available for members of the grass family. This work is identifying genes that are critical to development in *Arabidopsis* but were lost at some time before the diversification of grain crops.

As more expression and gene function data are being generated for a broader collection of plant species, phylogenetically-based comparative analyses will promote a deeper understanding of the molecular basis of variation in the development of flowers and other traits. Such advances will require careful staging of development in order to make appropriate cross-species comparisons (Buzgo *et al.*, 2004). Improved phylogenetic inferences of gene family evolution will require increased sampling of genes from species representing key lineages in the angiosperm phylogeny (Soltis *et al.*, 2004) and improved methods of automated sequence alignment for large and diverse gene families. Finally, phylogenetically-based methods have not yet been developed for identifying concerted changes in gene expression across the 100s of gene families represented in microarray studies. Resolving these issues in the near future will be a major breakthrough for the field of functional genomics.

### Acknowledgements

We thank all participants in the Plant Reproductive Genomics Workshop for a truly stimulating series of talks. Thanks go to Hong Ma and Günter Theissen for many discussions on the genetic control of floral development. Sangtae Kim provided Figure 2 depicting the ABC(DE) model. Travel costs for the workshop were funded in large part through National Science Foundation Plant Genome Grant, DBI-0115684, in support of the Floral Genome Project.

### References

Albert VA, Oppenheimer DG, Lindqvist C. 2002. Pleiotropy, redundancy and the evolution of flowers. *Trends Plant Sci* **7**: 297–301.

APGII. 2003. An update of the Angiosperm Phylogeny Group classification for the orders and families of flowering plants: APG II. *Bot J Linn Soc* **141**: 399–436.

Becker A, Theissen G. 2003. The major clades of MADS-box genes and their role in the development and evolution of flowering plants. *Mol Phylogenet Evol* **29**: 464–489.

Benedito VA, Visser PB, Angenent GC, Krens FA. 2004. The potential of virus-induced gene silencing for speeding up functional characterization of plant genes. *Genet Mol Res* **3**: 323–341.

Buzgo M, Soltis DE, Soltis PS, Ma H. 2004. Towards a comprehensive integration of morphological and genetic studies of floral development. *Trends Plant Sci* **9**: 164–173.

Buzgo M, Soltis PS, Soltis DE. 2005. Floral developmental morphology of *Amborella trichopoda* (Amborellaceae). *Int J Plant Sci* **165**: 925–947.

Caicedo AL, Stinchcombe JR, Olsen KM, Schmitt J, Purugganan MD. 2004. Epistatic interaction between *Arabidopsis* FRI and FLC flowering time genes generates a latitudinal cline in a life history trait. *Proc Natl Acad Sci USA* **101**: 15 670–15 675–.

Coen ES, Meyerowitz EM. 1991. The war of the whorls: genetic interactions controlling flower development. *Nature* **353**: 31–37.

Columbo L, Franken J, Koetje E, *et al*. 1995. The petunia MADS box gene FBP11 determines ovule identity. *Plant Cell* **7**: 1859–1868.

Davies B, Motte P, Keck E, *et al*. 1999. PLENA and FARINELLI: redundancy and regulatory interactions between two *Antirrhinum* MADS-box factors controlling flower development. *EMBO J* **18**: 4023–4034.

Ditta G, Pinyopich A, Robles P, Pelaz S, Yanofsky MF. 2004. The *SEP4* gene of *Arabidopsis thaliana* functions in floral organ and meristem identity. *Curr Biol* **14**: 1935–1940.

Doi K, Izawa T, Fuse T, *et al*. 2004. Ehd1, a B-type response regulator in rice, confers short-day promotion of flowering and controls FT-like gene expression independently of Hd1. *Genes Dev* **18**: 926–936.

Doust AN, Devos KM, Gadberry MD, Gale MD, Kellogg EA. 2004. Genetic control of branching in foxtail millet. *Proc Natl Acad Sci USA* **101**: 9045–9050.

Doust AN, Devos KM, Gadberry MD, Gale MD, Kellogg EA. 2005. The genetic basis for inflorescence variation between foxtail and green millet (Poaceae). *Genetics* (in press).

Doyle JJ. 1994. Evolution of a plant homeotic multigene family: toward connecting molecular systematics and molecular developmental genetics. *Syst Biol* **43**: 307–328.

Endress PK. 1997. Relationships between floral organization, architecture, and pollination mode in *Dillenia* (Dilleniaceae). *Plant Syst Evol* **206**: 99–118.

Endress PK. 2001. The flowers in extant basal angiosperms and inferences on ancestral flowers. *Int J Plant Sci* **162**: 1111–1140.

Fishman L, Kelly AJ, Willis JH. 2002. Minor quantitative trait loci underlie floral traits associated with mating system divergence in *Mimulus*. *Evolution* **56**: 2138–2155.

Fishman L, Willis JH. 2004. A novel meiotic drive locus near-completely distorts segregation in *Mimulus* (monkeyflower) hybrids. *Genetics* (in press).

Force A, Lynch M, Pickett FB, Amores A, Yan YL, Postlethwait J. 1999. Preservation of duplicate genes by complementary, degenerative mutations. *Genetics* **151**: 1531–1545.

Frohlich MW, Parker DS. 2000. The mostly male theory of flower evolutionary origins: from genes to fossils. *Syst Bot* **25**: 155–170.

Copyright © 2005 John Wiley & Sons, Ltd. 

Henikoff S, Till BJ, Comai L. 2004. TILLING. Traditional mutagenesis meets functional genomics. *Plant Physiol* **135**: 630–636.

Irish VF. 2003. The evolution of floral homeotic gene function. *Bioessays* **25**: 637–646.

Irish VF, Benfey PN. 2004. Beyond *Arabidopsis*. Translational biology meets evolutionary developmental biology. *Plant Physiol* **135**: 611–614.

Izawa T, Takahashi Y, Yano M. 2003. Comparative biology comes into bloom: genomic and genetic comparison of flowering pathways in rice and *Arabidopsis*. *Curr Opin Plant Biol* **6**: 113–120.

Jack T. 2004. Molecular and genetic mechanisms of floral control. *Plant Cell* **16**: S1–17.

Kim S, Koh J, Ma H, *et al*. 2005. Sequence and expression studies of A-, B-, and E-class MADS-box genes in *Eupomatia* (Eupomatiaceae): support for the bracteate origin of the calyptra. *Int J Plant Sci* (in press).

Kim S, Yoo M-J, Albert VA, *et al*. 2004. Phylogeny and diversification of B-function MADS-box genes in angiosperms: evolutionary and functional implications of a 260-million-year-old duplication. *Am J Bot* **91**: 2102–2118.

Kojima S, Takahashi Y, Kobayashi Y, *et al*. 2002. Hd3a, a rice orthologue of the *Arabidopsis* FT gene, promotes transition to flowering downstream of Hd1 under short-day conditions. *Plant Cell Physiol* **43**: 1096–1105.

Kramer EM, Dorit RL, Irish VF. 1998. Molecular evolution of genes controlling petal and stamen development: duplication and divergence within the APETALA3 and PISTILLATA MADS-box gene lineages. *Genetics* **149**: 765–783.

Kramer EM, Hall JC. 2005. Evolutionary dynamics of genes controlling floral development. *Curr Opin Plant Biol* **8**: 13–18.

Kramer EM, Jaramillo MA, Di Stilio VS. 2004. Patterns of gene duplication and functional evolution during the diversification of the AGAMOUS subfamily of MADS box genes in angiosperms. *Genetics* **166**: 1011–1023.

Kramer EM, Stilio VSD, Schluter PM. 2003. Complex patterns of gene duplication in the APETALA3 and PISTILLATA lineages of the Ranunculaceae. *Int J Plant Sci* **164**: 1–11.

Lamb RS, Irish VF. 2003. Functional divergence within the APETALA3/PISTILLATA floral homeotic gene lineages. *Proc Natl Acad Sci USA* **100**: 6558–6563.

Litt A, Irish VF. 2003. Duplication and diversification in the APETALA1/FRUITFULL floral homeotic gene lineage: implications for the evolution of floral development. *Genetics* **165**: 821–833.

Liu Y, Nakayama N, Schiff M, *et al*. 2004. Virus induced gene silencing of a DEFICIENS orthologue in *Nicotiana benthamiana*. *Plant Mol Biol* **54**: 701–711.

Malcomber ST, Kellogg EA. 2004. Heterogeneous expression patterns and separate roles of the SEPALLATA gene LEAFY HULL STERILE1 in grasses. *Plant Cell* **16**: 1692–1706.

Pelaz S, Ditta GS, Baumann E, Wisman E, Yanofsky MF. 2000. B and C floral organ identity functions require SEPALLATA MADS-box genes. *Nature* **405**: 200–203.

Pires JC, Zhao J, Schranz ME, *et al*. 2004. Flowering time divergence and genomic rearrangements in resynthesized *Brassica* polyploids (Brassicaceae). *Biol J Linn Soc* **82**: 409–700.

Purugganan MD, Rounsley SD, Schmidt RJ, Yanofsky MF. 1995. Molecular evolution of flower development: diversification of the plant MADS-box regulatory gene family. *Genetics* **140**: 345–356.

Robertson D. 2004. VIGS vectors for gene silencing: many targets, many tools. *Annu Rev Plant Biol* **55**: 495–519.

Schranz ME, Osborn TC. 2004. *De novo* variation in life-history traits and responses to growth conditions of resynthesized polyploid *Brassica napus* (Brassicaceae). *Am J Bot* **91**: 174–183.

Schwarz-Sommer Z, Huijser P, Nacken W, Saedler H, Sommer H. 1990. Genetic control of flower development by homeotic genes in *Antirrhinum majus*. *Science* **250**: 931–936.

Soltis DE, Albert VA, Savolainen V, *et al*. 2004. Genome-scale data, angiosperm relationships, and 'ending incongruence': a cautionary tale in phylogenetics. *Trends Plant Sci* **9**: 477–483.

Stefanovic S, Rice DW, Palmer JD. 2004. Long branch attraction, taxon sampling, and the earliest angiosperms: *Amborella* or monocots? *BMC Evol Biol* **4**: 35.

Stellari GM, Jaramillo MA, Kramer EM. 2004. Evolution of the APETALA3 and PISTILLATA lineages of MADS-box-containing genes in the basal angiosperms. *Mol Biol Evol* **21**: 506–519.

Stemple DL. 2004. TILLING — a high-throughput harvest for functional genomics. *Nature Rev Genet* **5**: 145–150.

Stinchcombe JR, Weinig C, Ungerer M, *et al*. 2004. A latitudinal cline in flowering time in *Arabidopsis thaliana* modulated by the flowering time gene FRIGIDA. *Proc Natl Acad Sci USA* **101**: 4712–4717.

Theissen G, Saedler H. 2001. Plant biology. Floral quartets. *Nature* **409**: 469–471.

Uimari A, Kotilainen M, Elomaa P, *et al*. 2004. Integration of reproductive meristem fates by a SEPALLATA-like MADS-box gene. *Proc Natl Acad Sci USA* **101**: 15 817–15 822–.

Vandenbussche M, Theissen G, Van de Peer Y, Gerats T. 2003. Structural diversification and neo-functionalization during floral MADS-box gene evolution by C-terminal frameshift mutations. *Nucleic Acids Res* **31**: 4401–4409.

Whipple CJ, Ciceri P, Padilla CM, *et al*. 2004. Conservation of B-class floral homeotic gene function between maize and *Arabidopsis*. *Development* **131**: 6083–6091.

Winter KU, Weiser C, Kaufmann K, *et al*. 2002. Evolution of class B floral homeotic proteins: obligate heterodimerization originated from homodimerization. *Mol Biol Evol* **19**: 587–596.

Zahn LM, Kong H, Leebens-Mack J, *et al*. 2005. The evolution of the SEPALLATA subfamily of MADS-box genes: a pre-angiosperm origin with multiple duplications throughout angiosperm history. *Genetics* (in press).

Zanis MJ, Soltis DE, Soltis PS, Mathews S, Donoghue MJ. 2002. The root of the angiosperms revisited. *Proc Natl Acad Sci USA* **99**: 6848–6853.

Copyright © 2005 John Wiley & Sons, Ltd. 
